# A genomic estimated breeding value-assisted reduction method of single nucleotide polymorphism sets: a novel approach for determining the cutoff thresholds in genome-wide association studies and best linear unbiased prediction

**DOI:** 10.1080/19768354.2023.2250841

**Published:** 2023-09-02

**Authors:** Young-Sup Lee, Jae-Don Oh, Jun-Yeong Lee, Donghyun Shin

**Affiliations:** a Department of Animal Biotechnology, Jeonbuk National University, Jeonju, Republic of Korea; b School of Life Sciences, BK21 FOUR KNU Creative BioResearch Group, Kyungpook National University, Daegu, Republic of Korea; c Department of Agricultural Convergence Technology, Jeonbuk National University, Jeonju, Republic of Korea

**Keywords:** Correlation difference (CD), genomic estimated breeding value (GEBV)-assisted reduction method of SNP set (GARS), landrace pigs, prediction ability (PA)

## Abstract

Traditionally, the *p*-value is the criterion for the cutoff threshold to determine significant markers in genome-wide association studies (GWASs). Choosing the best subset of markers for the best linear unbiased prediction (BLUP) for improved prediction ability (PA) has become an interesting issue. However, when dealing with many traits having the same marker information, the *p*-values’ themselves cannot be used as an obvious solution for having a confidence in GWAS and BLUP. We thus suggest a genomic estimated breeding value-assisted reduction method of the single nucleotide polymorphism (SNP) set (GARS) to address these difficulties. GARS is a BLUP-based SNP set decision presentation. The samples were Landrace pigs and the traits used were back fat thickness (BF) and daily weight gain (DWG). The prediction abilities (PAs) for BF and DWG for the entire SNP set were 0.8 and 0.8, respectively. By using the correlation between genomic estimated breeding values (GEBVs) and phenotypic values, selecting the cutoff threshold in GWAS and the best SNP subsets in BLUP was plausible as defined by GARS method. 6,000 SNPs in BF and 4,000 SNPs in DWG were considered as adequate thresholds. Gene Ontology (GO) analysis using the GARS results of the BF indicated neuron projection development as the notable GO term, whereas for the DWG, the main GO terms were nervous system development and cell adhesion.

## Introduction

Genome-wide association studies (GWAS) aim to detect variants of genomic loci associated with complex traits in the population, and common single nucleotide polymorphisms (SNPs) are the main source of these variants. SNPs are used to describe genetic variability and identify candidate genes, and GWAS examine significant SNPs (Lee et al. 2021; Lee et al. 2022). Attempts to use linkage analysis for mapping genomic loci that affect complex traits are ubiquitous. However, mapping of loci underlying complex traits has been less successful with linkage analysis. Thus, an association scan involving one million variants in the genome and a sample of unrelated individuals may be more powerful than linkage analysis with a few hundred markers (Visscher et al. 2012).

In animal breeding, best linear unbiased prediction (BLUP) is a technique used to estimate genetic ability. BLUP estimates the realized values of random variables (random effects) (Robinson 1991). Although BLUP usually uses the total SNPs as designed by SNP beadchips, some investigators have used part of the total SNPs when performing BLUP. They used this approach when the prediction of breeding values with SNP subset data provided better prediction ability (PA) (Li et al. 2018; Lu et al. 2020).

The Landrace pig is a long white pig variety utilized as the grandparents when producing F1 parent stock females in terminal crossbreeding. It outperforms other pigs in terms of litter size as well as birth and weaning weight. In Korea, Landrace pigs are typically used for commercial pork production (Lee and Shin 2018). Backfat thickness (BF) and daily weight gain (DWG) are important traits in pork production. The genomic regions affecting pork quality, such as BF and DWG, in Landrace and Duroc pigs were recently determined (Keel et al. 2020; Okumura et al. 2013; Rohrer et al. 2006).

We performed a genome-wide association study (GWAS) and the best linear unbiased prediction (BLUP) for BF and DWG. Determining significant markers in a GWAS allow the identification of notably associated genes and guide improved pig breeding programs. Furthermore, GWAS require multiple tests to determine significance. Currently, multiple hypothesis tests focus on the dependence among the hypotheses, specifically at the level of *p*-values calculated for multiple testing. Many theoretical and computational suggestions have been made for addressing *p*-value-related problems (Johnson et al. 2010; Storey 2011). Here, we devised a genomic estimated breeding value-assisted reduction method of SNP sets (GARS) as a different approach for determining the threshold. This approach is both data-driven and novel.

## Materials and methods

### Data preparation

We randomly sampled 2,933 Landrace pigs from various farms. Daily weight gain (DWG) and backfat thickness (BF) were analyzed. BF was measured from the longissimus dorsi muscle between the 10th and 11th ribs. The genomic DNA of Landrace pigs was genotyped using an Illumina Porcine 60 K SNP BeadChip (Illumina, San Diego, CA, USA) following the standard protocol. The total number of single nucleotide polymorphisms (SNPs) was 55,167; after quality control (QC), 46,992 SNPs remained in the autosomes (Chang et al. 2015). QC was performed using minor allele frequency (MAF < 0.05), genotyping rate threshold (< 0.05), and Hardy-Weinberg equilibrium (HWE), *P*-value < 1.0E-6. SNP genotypes were imputed using the Beagle program (Browning and Browning 2007).

### Preprocessing using linear regression (GWAS)

Linear regression was used to determine the association between genomic data and traits (Lee and Shin 2018). We conducted a linear regression analysis to determine the significance of each SNP. The GCTA program was used for the analysis, and the phenotypes used were BF and DWG (Yang et al. 2011). Pig sex and parity were included as covariates in the analysis.

### Genomic estimated breeding value-assisted reduction of SNPs (GARS)

Genomics Estimated breeding value-assisted reduction method of SNP sets (GARS) is used to determine an adequate number of SNPs. We determined the number of SNPs with significance in GWAS with the best prediction of breeding values in BLUP using GARS. The predictive ability (PA) correlation difference was calculated as follows:

(1) PA=cor(phenotypicvalues,breedingvalues)

(2) correlationdifference=PA[i∗step]−PA[(i−1)∗step]

where (i*step) represents the number of SNPs, and PA[i*step] indicates that it was derived from the SNP subset obtained from 1-i*step SNPs.

In our GWAS and BLUP analyses, 1,000 SNPs were used. The region immediately before the continuous negligible correlation difference was selected as the cut-off value. The R package ‘rrBLUP’ was used to determine the breeding values (Endelman 2011). The selected cutoff had the following criteria: (1) smaller than average correlation difference and (2) abrupt change in the correlation difference. This abrupt change indicated that the gene effect was considerably low and negligible. Additionally, the PA stability was evaluated using cross-validation (CV). This procedure involved dividing the dataset into training and test sets. To validate our results, K-fold CV (K = 10, test size = 0.3) was performed.

### Gene ontology (GO) analysis

Genes encompassing significant SNPs in the BF and DWG traits (GARS method) were further surveyed using GO analysis. The Ensembl database (www.ensembl.org) was used as the gene catalogue, and GO analysis was performed using the Database for Annotation, Visualization, and Integrated Discovery (DAVID, version 2021) (Huang et al. 2007). A list of gene identifiers was uploaded, and functional annotations were summarized. The GO results were based on the number of corresponding genes.

## Results

### Data description

**Figure 1.**
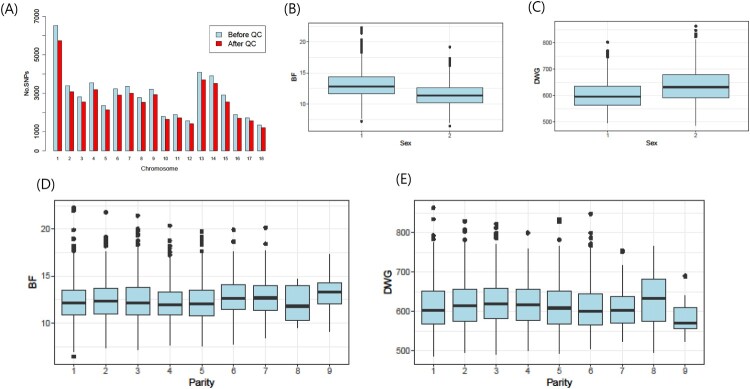
(A) Bar plot of the number of SNPs before and after quality control (QC) across autosomes. (B, C) Box plot of backfat thickness (BF) and daily weight gain (DWG) according to sex. (D, E) Box plot according to parity.

The numbers according to sex and parity in Landrace pigs and the mean and standard deviation (SD) of the traits are presented in Table 1. The mean distance (SD) between adjacent SNPs ranged from 40,630 (± SD 41,750; CHR 14) to 55,873 (± 73,708; CHR 15). The numbers of SNPs before and after QC across the autosomes (chromosomes 1–18) are shown in Figure 1(A). Boxplots of sex and parity in BF and DWG are also shown in Figure 1.

**Table 1.** Landrace pigs used in the study.

Sex	Male	Female
The number of Landrace pigs	1,667	1,266

**Table:** 

Parity	1	2	3	4	5	6	7	8	9
The number of Landrace pigs	698	551	534	472	368	184	85	24	17

**Table:** 

Trait	BF	DWG
Mean	12.4	618
SD	2.14	60
		

Note: BF, Backfat thickness; DWG, Daily weight gain.

### Results of the GARS method

**Figure 2.**
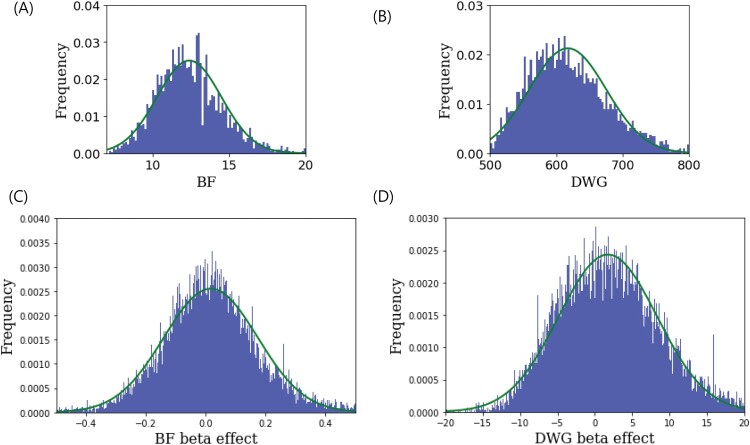
(A, B) Histograms of backfat thickness (BF) and daily weight gain (DWG)phenotypic values. (C, D) The histograms of beta effects in the genome-wide association study (GWAS) for BF and DWG. These showed that the phenotypic values and beta effects fit the normal curve (green).

In linear regression, the covariates used were sex and parity, and the phenotypes were BF and DWG. Histograms of phenotypic values and beta effects were plotted using a normal distribution curve (Figure 2). This illustrated the normality of the phenotypic values and beta effects. GARS was applied to GWAS and BLUP. To estimate breeding values, we used Genomic Best Linear Unbiased Prediction (G-BLUP). We observed the singularity of PA difference in GARS. (1) When the step was set to be tight at 50, PA estimation could lead to a very small number of SNPs as the cutoff value in the GWAS owing to a sudden decrease in PA difference. However, setting the step to 100 indicated that step 50 could not guarantee a steep change in the PA difference, suggesting that step 100 or larger is a reasonable choice. (2) Although the goals of GWAS and BLUP were disparate, we determined that the number of SNPs in the GWAS and BLUP were the same. One of the main strategies used in both studies was to determine the causative genomic regions or genes.

**Figure 3.**
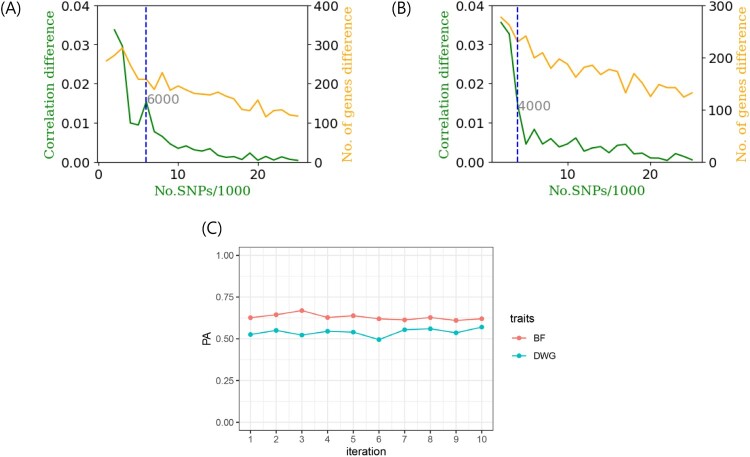
(A, B) The correlation difference (green) and number of gene differences (orange) against the number of single nucleotide polymorphisms (SNPs)/1,000 for backfat (BF) and daily weight gain (DWG), respectively. The cutoff was chosen as 6,000 (BF) and 4,000 (DWG) SNPs because these regions represented a larger correlation difference than the average (0.01), steep change and after that, a considerably negligible change. Number of newly added genes at each step (step: 1,000 SNPs) showed no greatly observable change. (C) The K-fold cross validation (CV, K = 10) of prediction ability (PA) for 6,000 and 4,000 SNPs in BF and DWG traits, respectively.

**Figure 4.**
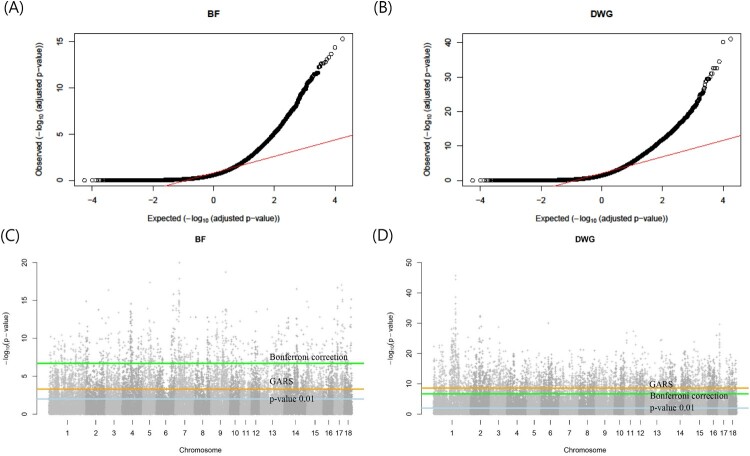
(A, B) The Quantile-Quantile plot (QQ plot) of the adjusted *p*-values in the genome-wide association study (GWAS) for backfat (BF) and daily weight gain (DWG), respectively. (C, D) Manhattan plot of the –log_10_(*p*-value) of GWAS (C: BF, D: DWG) across autosomes in Landrace pigs. The three horizontal lines represent the three kinds of cutoff (light blue: *p*-value 0.01, orange: genomic estimated breeding value-assisted reduction method of the single nucleotide polymorphism set (GARS)method, green: Bonferroni correction (corrected *p*-value 0.01)). The GARS method was more congenital than Bonferroni correction for BF and was harsher for DWG.

Figure 3 shows the correlation difference defined by Equation (1) and the number of gene differences with steps of 1,000 in the BF and DWG. Under the absence of considerable number of gene differences with increasing number of SNPs, the differences in PA showed noticeable changes (Figure 3(A) and 1(B)). The remaining SNPs, except for those chosen in the GARS results, were highly negligible in both BF and DWG. Thus, we conclude that an adequate 6,000 and 4,000 SNPs were adequate for the cut-off in BF and DWG, respectively. The PAs using total SNPs and chosen SNP subset were 0.8 (BF), 0.81 (DWG), and 0.74 (BF; 6,000 SNPs), 0.73 (DWG; 4,000 SNPs). The number significant genes obtained with GARS and Bonferroni correction were 1,521 and 318 in BF, respectively. In DWG, 1,027 and 1,540 significant genes were obtained, respectively. The estimated heritabilities were 0.3 (standard error:0.2) and 0.3 (0.007) in BF and DWG, respectively. These results are similar to those of a previous study on Duroc pigs (0.37 and 0.34 in BF and DWG, respectively) (Herrera-Cáceres et al. 2022). The selected SNP subset was cross-validated further (Figure 3(C)). The mean and SD of PA for BF and DWG in the K-fold CV (K = 10) were 0.63 (SD: ± 0.02) and 0.53 (± 0.02), respectively. The Q-Q plots are shown in Figure 4(A) and 4(B). The Q-Q plot of *p*-values confirmed the GWAS results. The *p*-values were adjusted using the Benjamini-Hochberg correction in the Q-Q plot.

### A comparison of GARS with the traditional methods

In association studies, the False Discovery Ratio (FDR), Benjamini-Hochberg (BH) method, and Bonferroni correction were used to identify significant variants. In the Bonferroni correction, the *p*-value was adjusted according to the sample size. In contrast, GARS and the statistic used (Equation (2)) are heuristic. Using GARS, we determined the cut-off thresholds for GWAS and BLUP. For BF and DWG, the Manhattan plot showed that Bonferroni correction resulted in a common cutoff *p*-value of 2.0*E-7, but respective *p*-values of 5.0*E-4 (6,000 SNPs in BF) and 2.5*E-9 (4,000 SNPs in DWG), respectively (Figure 4(C) and 4(D)). The Bonferroni correction was harsher than GARS for the BF results, whereas GARS was harsher than Bonferroni correction for the DWG results. Compared with other methods, GARS is a more data-driven method. Thus, GARS can provide flexible cutoffs based on the traits of interest.

### Gene ontology (GO) analysis of significant SNPs

The highly significant SNPs and encompassing genes in the GWA test were demonstrated with gene ontologies (GO, respectively, of Biological Process (BP) and molecular function [MF]) (Supplementary Data 1,2). We also determined whether GOs for BF and DWG overlapped.

According to GO analysis (*p*-value < 1.0E-06) using the BF-associated genes, neuron projection development was a notable BP term, and ion binding was a notable MF term. The notable GOs for DWG (*p* < 1.0E-05) were nervous system development, cell adhesion (BP), and ion binding (MF) (Supplementary Data 3, 4). Among the 19 top GO terms for BF, eight overlapped with the Bonferroni correction results (e.g. GO:0030182∼neuron differentiation). In addition, 12 of the 16 GO terms in the DWG overlapped with Bonferroni correction (GO:0007399, nervous system development; GO:0007155, cell adhesion).

## Discussion

In GWAS, *p*-values or adjusted *p*-values, such as the Bonferroni correction or false discovery ratio (FDR), have been used for association tests (Glickman et al. 2014; VanderWeele and Mathur 2019). However, even the Bonferroni correction and FDR exploit the *p*-value, principally considering the sample size. In the traditional Best Linear Unbiased Prediction (BLUP), regression using total SNPs is prevalent. Recently, BLUP has been performed using a subset of SNPs (Bermingham et al. 2015; Lee et al. 2015). However, a crucial problem with BLUP using a subset of SNPs is that it does not provide an appropriate number of SNPs. GARS determines how to divide SNPs into significant and non-significant ones. It can be applied to GWAS and BLUP, which aim to identify significantly associated SNPs and determine the best prediction of breeding value, respectively. GARS and the statistics used (Equation (2)) are somewhat heuristic, and the procedures can be demanding (depending on how the step set). However, the GARS approach permits any BLUP method that can provide the genomic estimated breeding values (GEBVs) and traits of interest.

Notable genes in the GOs of BF included Laminin subunit gamma 2 (LAMC2), Xenotropic and polytropic retrovirus receptor 1 (XPR1), and Neuregulin 3 (NRG3; overlapped with the GO of DWG). Previous studies have reported that upregulated LAMC2 activates CD44 and positively regulates docosanoic acid, palmitic acid, and trans-oleic acid content, while negatively regulating tridecanoic acid, stearic acid, and cis-5,8 11, 14-eicosapentaenoic acid contents (Liu et al. 2020). The biological roles of inorganic phosphate require careful regulation of its transport across cell membranes. The major cellular phosphate export protein, XPR1, is regulated by the most energetic cell signaling molecule, 1,5-bisdiphosphoinositol 1,2,3,4-tetrakisphosphate (InsP_8_) (Li et al. 2020). Neuregulin 3 (NRG3) is a member of the neuregulin gene family. This gene encodes ligands for the transmembrane tyrosine kinase receptors ERBB3 and ERBB4, which are members of the epidermal growth factor receptor family. Ligand binding activates intracellular signaling cascades and induces cellular responses, including proliferation, differentiation, migration, survival, and apoptosis (Genecards. org).

The observable genes in the GO of DWG were Insulin-like growth factor 1 receptor (IGF1R), Rubicon autophagy regulator (RUBCN), and FERM domain-containing 5 (FRMD5). The IGFs system is a family that includes two ligands (IGF-1 and IGF-2), two cell surface receptors (IGF1R and IGF2R), and at least six high-affinity IGF-binding proteins (IGFBPs 1-6). This system plays an essential role in normal human and animal development, including embryogenesis, pre- and postnatal growth, and maintenance of tissue homeostasis (Pan et al. 2012). The RUBCN gene is a negative regulator of autophagy and endocytic trafficking, and controls endosome maturation. The two conserved domains of this protein are the N-terminal RUN domain and the C-terminal DUR4206 domain. The N-terminal RUN domain is involved in Ras-like GTPase signaling, and the C-terminal DUF4206 domain contains a diacylglycerol (DAG) binding-like motif. Alternatively, spliced transcript variants encoding distinct isoforms have been identified. FRMD5 enables integrin and protein kinase-binding activities. It is involved in the negative regulation of cell motility and positive regulation of cell adhesion and regulation of cell migration. It is located at the adherens junction (genecards.org).

## Conclusion

The genomic estimated breeding value (GEBV)-assisted reduction of single nucleotide polymorphism (SNP) sets (GARS) is an alternative to traditional methods. The correlation difference (CD) predicted between GEBV and phenotypic values can guide the cutoff thresholds in GWAS and BLUP analyses. We observed a decreased CD in some SNP subsets and determined that the subset containing more SNPs was negligible because of the rapid loss of capability as a significant variant. The results of GARS and Bonferroni correction differed from each other, such that GARS was more congenital than Bonferroni correction in the BF trait, but was harsher in the DWG trait. Despite being a novel approach, the GARS results can be limited by extant association studies. However, we expect that GARS will play an important role in determining the number of SNPs and genes in GWAS and BLUP.

## Supplementary Material

Supplemental Material
