# Supplementary material for: A genomic estimated breeding value-assisted reduction method of single nucleotide polymorphism sets: a novel approach for determining the cutoff thresholds in genome-wide association studies and best linear unbiased prediction
Source: Anim Cells Syst (Seoul). 2023 Sep 2;27(1):180–6. doi: 10.1080/19768354.2023.2250841 (PMC10478620; doi:10.1080/19768354.2023.2250841)
Supplement: Supplemental Material [file TACS_A_2250841_SM2379.zip › Supplementary Data 1.docx]

Supplementary Data 1. The most significant single nucleotide polymorphisms (SNPs) and those encompassing genes associated with backfat (BF).

| **CHR** | **SNP** | **Position** | **GO** | **Gene** | **p-value** | **GO overlap*** | **Category** |
| --- | --- | --- | --- | --- | --- | --- | --- |
| 17 | ALGA0093899 | 27,606,083 | GO:0005216, ion channel activity | SLC24A3 | 2.07E-17 | No | MF |
| 14 | ASGA0064628 | 83,958,249 | GO:0050767, regulation of neurogenesis | NRG3 | 3.07E-17 | Yes | BP (BF), MF (DWG) |
| 18 | ASGA0080142 | 51,644,236 | GO:0010975, regulation of neuron projection development | HECW1 | 9.87E-15 | No | BP |
| 9 | DRGA0009770 | 124,492,658 | GO:0007399, nervous system development | LAMC2 | 1.49E-14 | No | BP |
| 9 | H3GA0028058 | 122,404,973 | GO:0043167, ion binding | XPR1 | 1.83E-14 | No | MF |
| 6 | ALGA0117399 | 141,280,500 | GO:0010975, regulation of neuron projection development | NEGR1 | 1.93E-14 | No | BP |
| 4 | ALGA0025280 | 63,108,540 | GO:0022836, gated channel activity | KCNB2 | 6.75E-14 | No | MF |
| 7 | ASGA0031851 | 23,254,681 | GO:0007399, nervous system development | FLOT1 | 1.36E-13 | No | BP |
| 8 | MARC0038361 | 126,024,923 | GO:0010975, regulation of neuron projection development (BP),  GO:0022836, gated channel activity (MF) | GRID2 | 1.75E-13 | No | BP, MF |
| 17 | ALGA0093898 | 27,528,186 | GO:0005216, ion channel activity | SLC24A3 | 5.13E-13 | No | MF |
| 4 | ASGA0019791 | 63,054,580 | GO:0022836, gated channel activity | KCNB2 | 5.74E-13 | No | MF |

*GO overlap: overlapped or not with daily weight gain (DWG) GO terms.

** The p-value of gene ontology (GO) terms was below 1.0E-06 and the categories (biological process (BP) and molecular function (MF)) were shown. The GO terms can be referenced to in Supplementary Data 3.
