# Supplementary material for: A genomic estimated breeding value-assisted reduction method of single nucleotide polymorphism sets: a novel approach for determining the cutoff thresholds in genome-wide association studies and best linear unbiased prediction
Source: Anim Cells Syst (Seoul). 2023 Sep 2;27(1):180–6. doi: 10.1080/19768354.2023.2250841 (PMC10478620; doi:10.1080/19768354.2023.2250841)
Supplement: Supplemental Material [file TACS_A_2250841_SM2379.zip › Supplementary Data 2.docx]

Supplementary Data 2. The most significant single nucleotide polymorphisms (SNPs) and those encompassing genes associated with daily weight gain (DWG).

| **CHR** | **SNP** | **Position** | **GO** | **Gene** | **p-value** | **GO overlap*** | **Category** |
| --- | --- | --- | --- | --- | --- | --- | --- |
| 1 | H3GA0002645 | 130,108,758 | GO:1902580, single-organism cellular localization (BP),  GO:0043167, ion binding (MF) | CHP1 | 9.14E-29 | No | BP, MF |
| 13 | ALGA0071900 | 134,306,077 | GO:0043167, ion binding | RUBCN | 4.70E-25 | Yes | MF (BF), MF (DWG) |
| 1 | MARC0103956 | 138,369,258 | GO:0000902, cell morphogenesis (BP),  GO:0005488, binding (MF) | MEF2A | 5.44E-25 | Yes | BP (BF, DWG), MF (BF, DWG) |
| 1 | MARC0001422 | 140,492,801 | GO:0007399, nervous system development | GABRA5 | 1.11E-24 | No | BP |
| 15 | ASGA0102804 | 21,301,002 | GO:1902580, single-organism cellular localization (BP),  GO:0005488, binding (MF) | DPP10 | 1.42E-24 | Yes | BP, MF |
| 1 | ASGA0096650 | 137,394,981 | GO:0043167, ion binding | IGF1R | 1.03E-23 | No | MF |
| 1 | ALGA0003091 | 52,127,592 | GO:1902580, single-organism cellular localization (BP),  GO:0043167, ion binding (MF) | RIMS1 | 1.80E-23 | Yes | BP (BF, DWG), MF (BF, DWG) |
| 1 | H3GA0002668 | 132,999,230 | GO:0005488, binding | SPRED1 | 1.14E-22 | No | MF |
| 15 | ASGA0100769 | 21,207,190 | GO:1902580, single-organism cellular localization (BP),  GO:0005488, binding (MF) | DPP10 | 3.15E-22 | Yes | BP, MF |
| 1 | MARC0037875 | 127,469,701 | GO:0007155, cell adhesion (BP),  GO:0005488, binding (MF) | FRMD5 | 3.98E-22 | No | BP, MF |
| 3 | ALGA0114825 | 79,728,804 | GO:0000902, cell morphogenesis (BP),  GO:0003824, catalytic activity (MF) | B3GNT2 | 6.68E-22 | No | BP, MF |

*GO overlap: overlapped or not with back fat (BF) GO terms.

**The p-value of gene ontology (GO) terms was below 1.0E-05. The GO terms can be referenced to Supplementary Data 4.
