# Supplementary material for: A genomic estimated breeding value-assisted reduction method of single nucleotide polymorphism sets: a novel approach for determining the cutoff thresholds in genome-wide association studies and best linear unbiased prediction
Source: Anim Cells Syst (Seoul). 2023 Sep 2;27(1):180–6. doi: 10.1080/19768354.2023.2250841 (PMC10478620; doi:10.1080/19768354.2023.2250841)
Supplement: Supplemental Material [file TACS_A_2250841_SM2379.zip › Supplementary Data 3.docx]

Supplementary Data 3. The Gene Ontology (GO) analysis using genome-wide association (GWA) test results for backfat (BF) (GO p-value < 1.0E-05).

| **Category** | **Term** | **Count** | **p-value** | **Fold Enrichment** |
| --- | --- | --- | --- | --- |
| BP | GO:0010975, regulation of neuron projection development | 63 | 7.77E-13 | 2.68 |
| BP | GO:0045664, regulation of neuron differentiation | 75 | 7.77E-12 | 2.32 |
| BP | GO:0050767, regulation of neurogenesis | 86 | 2.11E-10 | 2.04 |
| BP | GO:0007399, nervous system development | 200 | 4.84E-10 | 1.52 |
| BP | GO:0051960, regulation of nervous system development | 94 | 5.91E-10 | 1.93 |
| BP | GO:0048468, cell development | 196 | 2.67E-09 | 1.50 |
| BP | GO:0030030, cell projection organization | 145 | 2.75E-09 | 1.63 |
| BP | GO:0031344, regulation of cell projection organization | 72 | 4.16E-09 | 2.07 |
| BP | GO:0060284, regulation of cell development | 94 | 1.38E-08 | 1.82 |
| BP | GO:0022008, neurogenesis | 150 | 1.72E-08 | 1.57 |
| BP | GO:0048666, neuron development | 107 | 2.02E-08 | 1.73 |
| BP | GO:0048858, cell projection morphogenesis | 70 | 2.78E-08 | 2.00 |
| BP | GO:0030182, neuron differentiation | 127 | 3.11E-08 | 1.63 |
| BP | GO:0031175, neuron projection development | 90 | 4.39E-08 | 1.80 |
| BP | GO:0048812, neuron projection morphogenesis | 68 | 4.82E-08 | 2.00 |
| BP | GO:0032990, cell part morphogenesis | 71 | 5.34E-08 | 1.96 |
| BP | GO:0050773, regulation of dendrite development | 24 | 7.23E-08 | 3.61 |
| BP | GO:0048699, generation of neurons | 138 | 1.12E-07 | 1.55 |
| BP | GO:0010976, positive regulation of neuron projection development | 33 | 2.55E-07 | 2.73 |
| MF | GO:0043167, ion binding | 496 | 5.39E-10 | 1.25 |
| MF | GO:0022836, gated channel activity | 56 | 5.53E-09 | 2.32 |
| MF | GO:0005216, ion channel activity | 64 | 2.38E-08 | 2.10 |
| MF | GO:0022838, substrate-specific channel activity | 65 | 2.95E-08 | 2.07 |
| MF | GO:0043169, cation binding | 324 | 4.76E-08 | 1.30 |
| MF | GO:0046872, metal ion binding | 317 | 6.27E-08 | 1.31 |
| MF | GO:0015267, channel activity | 66 | 1.54E-07 | 1.97 |
| MF | GO:0022803, passive transmembrane transporter activity | 66 | 1.54E-07 | 1.97 |
| MF | GO:0005509, calcium ion binding | 85 | 4.05E-07 | 1.75 |

* The analyzed cutoff of the single nucleotide polymorphisms (SNPs) was determined using genomic estimated breeding value-assisted reduction method of the SNP set (GARS). Neuron projection development (BP) and ion binding (MF) were the notable GOs.
