# Supplementary material for: A genomic estimated breeding value-assisted reduction method of single nucleotide polymorphism sets: a novel approach for determining the cutoff thresholds in genome-wide association studies and best linear unbiased prediction
Source: Anim Cells Syst (Seoul). 2023 Sep 2;27(1):180–6. doi: 10.1080/19768354.2023.2250841 (PMC10478620; doi:10.1080/19768354.2023.2250841)
Supplement: Supplemental Material [file TACS_A_2250841_SM2379.zip › Supplementary Data 4.docx]

Supplementary Data 4. Gene Ontology (GO) analysis using the genome-wide association (GWA) test results for daily weight gain (DWG) (GO p-value < 1.0E-05).

| **Category** | **Term** | **Count** | **p-value** | **Fold Enrichment** |
| --- | --- | --- | --- | --- |
| BP | GO:1902580, single-organism cellular localization | 76 | 1.86E-08 | 2.0 |
| BP | GO:0000902, cell morphogenesis | 76 | 1.40E-07 | 1.9 |
| BP | GO:0051650, establishment of vesicle localization | 31 | 3.86E-07 | 2.8 |
| BP | GO:0051648, vesicle localization | 31 | 6.78E-07 | 2.8 |
| BP | GO:0007399, nervous system development | 131 | 1.73E-06 | 1.5 |
| BP | GO:0050767, regulation of neurogenesis | 55 | 2.45E-06 | 2.0 |
| BP | GO:0010975, regulation of neuron projection development | 37 | 2.46E-06 | 2.4 |
| BP | GO:0032989, cellular component morphogenesis | 77 | 3.23E-06 | 1.7 |
| BP | GO:0022604, regulation of cell morphogenesis | 40 | 3.74E-06 | 2.2 |
| BP | GO:0045664, regulation of neuron differentiation | 45 | 4.65E-06 | 2.1 |
| BP | GO:0051960, regulation of nervous system development | 60 | 5.09E-06 | 1.9 |
| BP | GO:0051640, organelle localization | 48 | 5.29E-06 | 2.0 |
| BP | GO:0051656, establishment of organelle localization | 42 | 5.49E-06 | 2.1 |
| BP | GO:0007155, cell adhesion | 92 | 8.42E-06 | 1.6 |
| BP | GO:0097480, establishment of synaptic vesicle localization | 21 | 9.59E-06 | 3.1 |
| BP | GO:0048489, synaptic vesicle transport | 21 | 9.59E-06 | 3.1 |
| MF | GO:0043167, ion binding | 347 | 3.79E-11 | 1.3 |
| MF | GO:0043168, anion binding | 195 | 7.86E-10 | 1.5 |
| MF | GO:0005488, binding | 637 | 2.14E-09 | 1.1 |
| MF | GO:0032559, adenyl ribonucleotide binding | 121 | 3.46E-09 | 1.7 |
| MF | GO:0030554, adenyl nucleotide binding | 121 | 5.27E-09 | 1.7 |
| MF | GO:0032553, ribonucleotide binding | 143 | 7.87E-09 | 1.6 |
| MF | GO:0005524, ATP binding | 117 | 1.68E-08 | 1.7 |
| MF | GO:0032555, purine ribonucleotide binding | 140 | 2.49E-08 | 1.6 |
| MF | GO:1901265, nucleoside phosphate binding | 154 | 4.44E-08 | 1.5 |
| MF | GO:0000166, nucleotide binding | 154 | 4.44E-08 | 1.5 |
| MF | GO:0017076, purine nucleotide binding | 140 | 4.45E-08 | 1.6 |
| MF | GO:0036094, small molecule binding | 172 | 4.65E-08 | 1.5 |
| MF | GO:0035639, purine ribonucleoside triphosphate binding | 136 | 7.55E-08 | 1.6 |
| MF | GO:0016772, transferase activity, transferring phosphorus-containing groups | 84 | 1.54E-07 | 1.8 |
| MF | GO:0097367, carbohydrate derivative binding | 153 | 1.64E-07 | 1.5 |
| MF | GO:0016301, kinase activity | 74 | 2.67E-07 | 1.9 |
| MF | GO:0016773, phosphotransferase activity, alcohol group as acceptor | 64 | 7.38E-07 | 1.9 |
| MF | GO:0003824, catalytic activity | 353 | 1.93E-06 | 1.2 |
| MF | GO:0016740, transferase activity | 158 | 9.17E-06 | 1.4 |

* The analyzed cutoff of the single nucleotide polymorphisms (SNPs) was determined using genomic estimated breeding value-assisted reduction method of the SNP set (GARS). The notable GOs were nervous system development and cell adhesion (BP) and ion binding (MF).
